# Current Evidence on the Involvement of RAGE–Diaph1 Signaling in the Pathology and Treatment of Neurodegenerative Diseases—An Overview

**DOI:** 10.3390/pathophysiology32030043

**Published:** 2025-08-29

**Authors:** Judyta K. Juranek, Bernard Kordas, Piotr Podlasz, Agnieszka Bossowska, Marta Banach

**Affiliations:** 1 Department of Human Physiology and Pathophysiology, School of Medicine, University of Warmia and Mazury in Olsztyn, 11-041 Olsztyn, Poland; bernard.kordas@uwm.edu.pl (B.K.); agnieszka.bossowska@uwm.edu.pl (A.B.); 2 Department of Pathophysiology, Forensic Veterinary Medicine and Administration, Faculty of Veterinary Medicine, University of Warmia and Mazury in Olsztyn, 10-719 Olsztyn, Poland; piotr.podlasz@uwm.edu.pl; 3 Department of Neurology, Collegium Medicum, Jagiellonian University, 31-530 Kraków, Poland

**Keywords:** neurodegenerative diseases, receptor for advanced glycation end-products, Diaph1, pathogenesis, treatment, signaling, neurodegeneration

## Abstract

Neurodegenerative diseases are a group of disorders characterized by the progressive deterioration of the structure and function of central nervous system neurons and include, among others, amyotrophic lateral sclerosis (ALS), multiple sclerosis (MS), Parkinson’s (PD), Alzheimer’s (AD), and Huntington’s (HD) diseases. And while all these diseases seem to have different genetic and environmental components, growing evidence shows that they share common underlying pathological features such as increased neuroinflammation and excessive oxidative stress. RAGE, the receptor for advanced glycation end-products, is a signal transduction receptor, and its activation triggers an increase in proinflammatory molecules, oxidative stressors, and cytokines. Diaph1, protein diaphanous homolog 1, is an actin modulator and an intracellular ligand of RAGE. Studies demonstrated that RAGE and Diaph1 act together, and their downstream signaling pathways play a role in neurodegeneration. Here, based on current evidence and our own research, we provide an overview of the RAGE–Diaph1 signaling and discuss the therapeutic potential of targeted therapy aimed at RAGE–Diaph1 signaling inhibition in the prevention and treatment of neurodegenerative diseases.

## 1. Introduction

Neurodegenerative diseases are a group of disorders characterized by the progressive deterioration of the structure and function of central nervous system neurons. Commonly, the group includes diseases of both brain and spinal cord neurons, such as amyotrophic lateral sclerosis (ALS), multiple sclerosis (MS), Parkinson’s (PD), Alzheimer’s (AD), and Huntington’s (HD) diseases [1]. Currently, with the exception of multiple sclerosis, there has been no successful treatment for any of the diseases, making them a priority both in the private and public neuromedical sectors. Providing effective treatments for any of these diseases is extremely important, considering an almost exponential growth in the number of neurodegenerative disease cases.

It is estimated that AD and PD will surpass cancer-related deaths by 2050 [2], and while these are the most common among neurodegenerative disorders, the others are becoming more frequent as the world population grows, and an increasing number of people are at risk. And while all these diseases seem to have different genetic and environmental components, growing evidence shows that they share common underlying pathological features such as increased neuroinflammation and excessive oxidative stress [3].

RAGE, the receptor for advanced glycation end-products, is a part of the immunoglobulin family and participates in the first line of the body’s defense system. It acts as a pattern recognition receptor (PRR) that helps detect damage-associated molecular patterns (DAMPs), initiating appropriate immune and repair responses. It is a signal transduction receptor, and its activation triggers an increase in proinflammatory molecules, oxidative stressors, and cytokines [4]. RAGE was first identified as a cell surface receptor for Advanced Glycation End-products (AGEs) [5,6], the products of nonenzymatic glycation and oxidation of proteins/lipids that accumulate during physiological aging, but also in diabetes, inflammatory, and neurodegenerative diseases.

**Figure 1:**
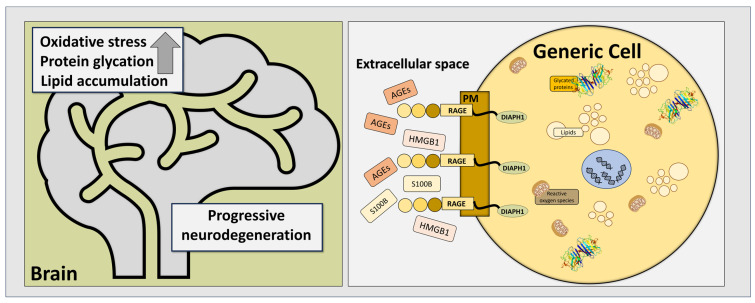
RAGE–ligand interactions play a significant role in many biochemical pathways involved in the pathogenesis of neurodegenerative diseases. Upregulation in levels of AGEs, as well as in soluble forms of S100B and HMGB1, activates RAGE and triggers its conformational changes. These changes lead to its binding to Diaph1 and cause further molecular events in affected cells, resulting in their dysfunction and, finally, death. PM—plasma membrane; generic cell represents both neuroglial cells, astrocytes, oligodendrocytes, ependymal cells, and microglia, as well as neuronal cells (motor, sensory, and interneurons).

Diaph1, protein diaphanous homolog 1, is a member of the Rho-GTPase formins [7], primarily involved in the modulation of cytoskeleton proteins, regulating cellular morphology, motility, and secretion. Studies demonstrated that RAGE and Diaph1 act together, with RAGE binding to Diaph1 via its intracellular domain [8], and their downstream signaling pathways are a part of biochemical cascades involved in neurodegeneration, see Figure 1.

## 2. RAGE and Its Most Prominent Ligands in Neurodegenerative Disorders

### 2.1. RAGE

In humans, the gene encoding RAGE, *AGER*, is located on chromosome six, within the MHC (major histocompatibility complex) region. RAGE has several different isoforms, resulting from alternative RNA splicing, and exists in two primary forms: full-length, membrane-bound (mRAGE) and extracellular, soluble (sRAGE) form. Physiologically, RAGE expression is only enriched in pneumocytes and endothelial cells; however, it increases in a disease state, in response to elevated levels of its extracellular ligands, such as AGEs, HMGB1 (high mobility group box 1 protein), or a calcium-binding protein B (S100B) [5,9].

Although RAGE signaling plays a physiological role in maintaining tissue homeostasis during stress responses, its chronic or dysregulated activation can have detrimental consequences. In many neurodegenerative and inflammatory disorders, sustained RAGE activation fuels a cycle of inflammation and oxidative stress, overriding its initial protective role. This pathological shift contributes to progressive neuronal damage and impaired resolution of inflammation. Elevated expression of RAGE has been demonstrated in multiple conditions and disorders, including neurodegenerative diseases [10].

Mechanistically, RAGE interacts with its extracellular ligands, triggering downstream cascades that lead to cytokine production, reactive oxygen species (ROS) generation, and cellular dysfunction. In acute scenarios, these responses may support repair, but when prolonged, they lead to cellular dysfunction and contribute to cell and tissue deterioration, triggering multiple symptoms and worsening the disease [10].

### 2.2. HMGB1

HMGB1 protein, also known as amphoterin, is encoded by the *HMGB1* gene located on chromosome 13 [11,12]. Similarly to RAGE, it has several isoforms and two forms: nuclear and extracellular. HMGB1 is one of the most important chromatin-associated proteins, acting as a DNA chaperone [13] [Human Protein Atlas: proteinatlas.org, https://www.proteinatlas.org/ version 24.0, accessed on 29 June 2025]. Similarly to RAGE, it is also involved in an innate immune response, acting as a proinflammatory cytokine and triggering an immune reaction [14] via binding to RAGE or TLR4 (toll-like receptor 4) and, as such, contributing to the pathogenesis of neurodegenerative disorders. In our research, we demonstrated that HMGB1 was elevated in the spinal cord of deceased ALS patients, underlying its likely involvement in ALS pathogenesis [15]. In addition, an increased expression of HMGB1 was also noted in HD, and its presence was implied to contribute to the accelerated rate of huntingtin accumulation in HD [16].

Interestingly though, evidence from studies on spinocerebellar ataxia type 1 (SCA1)—a rare autosomal dominant neurodegenerative disease—showed that the reduction in HMGB1 expression was detrimental in the SCA1 mouse model, while its overexpression restored DNA damage in affected cerebellar Purkinje cells and spinal cord motor neurons, improving motor function and prolonging the life of SCA1 mice [17]. On the contrary, in AD, the inhibition of HMGB1 with anti-HMGB1 antibodies was beneficial, halting axonal degeneration and contributing to improved cognition in a mouse model of AD [18].

### 2.3. S100B

S100B, located on chromosome 21 in humans, belongs to a larger family of calcium-binding regulatory S100 proteins. It is primarily intracellular; however, similarly to RAGE and HMGB1, it also has an extracellular form released by astrocytes and involved in neuroinflammation [19]. Increased cerebrospinal fluid levels of S100B have also been reported in PD [20], AD [21], and schizophrenia [22], implying roles for S100B in the pathogenesis of neurodegenerative diseases. In AD, RAGE plays a critical role in Aβ clearance, while in PD, the ablation of S100B leads to neuroprotection, reduced microgliosis, and decreased expression of both RAGE and TNFα. In ALS samples obtained from human patients, increased expression of S100B was observed in cortical and spinal cord astrocytes as well as in spinal cord neurons, while in rodent models, its increased expression was only observed in astrocytes but not in neurons [23,24,25,26,27,28,29]. In ALS tissues, RAGE increases in cells resembling astrocytes and microglia when motor neurons are lost, while total RAGE mRNA levels remain stable. In the mouse model of ALS, RAGE staining is visible only in affected animals, without specific cellular localization.

ALS patients exhibit increased S100B immunoreactivity in the brain and spinal cord, with varying localization: clustered astrocytes in the primary motor cortex or both astrocytes and motor neurons in the spinal cord. A rat model of ALS identified S100B in a subpopulation of glial fibrillary acidic protein (GFAP)-positive astrocytes labeled with Ki67 and characterized by increased toxicity towards motor neurons [15,24,25,29,30].

### 2.4. Diaph1—An Intracellular Protein with RAGE-Binding Domain

Diaph1 is a cytoplasmic protein and, together with Diaph2 and Diaph3, belongs to a family of Diaphanous-related formins (DRFs) [31]. It is encoded by the *DIAPH1* gene located on chromosome 5 [32] and has 14 splice variants. Mutations in the *DIAPH1* gene cause autosomal dominant non-syndromic sensorineural deafness with or without thrombocytopenia [33,34]. At the same time, the loss of the *DIAPH1* gene is associated with an autosomal recessive neurodevelopmental disorder known as seizures, cortical blindness, and microcephaly syndrome (SCBMS) [35] alone or combined with immunodeficiency and mitochondrial dysfunction [36].

Studies demonstrated that in ALS-linked Profilin1 variants, Diaph1 expression is enriched, altering formin-induced actin polymerization in the presence of its mutated ligand Profilin1 C71G variant in cell cultures [37]. Furthermore, it was also shown in the ALS mouse model that alterations in Diaph1 coding itself are predicted to be deleterious and detrimental to motor neuron survival in ALS [38]. The interaction between RAGE and Diaph1 was discovered and first described by Schmidt and coworkers in 2008 [39]. In the paper, the authors described in detail the binding mechanisms between RAGE and Diaph1, paving the way for further pre-clinical and pharmaceutical research. The study has proved to be crucial in terms of deciphering RAGE transduction pathways and demonstrated that for RAGE to effectively transduce the signal, it must bind to Diaph1, while Diaph1 may carry out its functions effectively either together or independently of RAGE. Since the time of the discovery, a large progress has been made in discerning the role of RAGE–Diaph1 signaling in neuroinflammation and hyperglycemia, and a number of small-molecule RAGE–Diaph1 inhibitors have been patented [40] allowing for both experimental and clinical studies.

**Figure 2:**
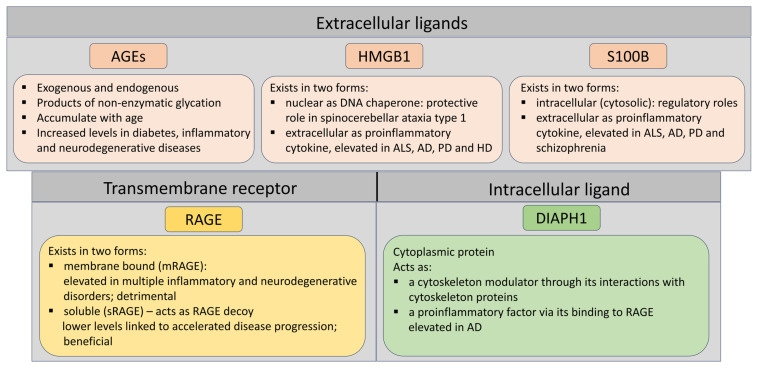
Diagram summarizing the roles of RAGE and its most prominent ligands in neurodegenerative diseases as mentioned in the text.

See Figure 2 for a graphical summary.

## 3. RAGE–Diaph1 Proteomic and Transcriptomic Expression Patterns in Neurodegenerative Disorders

### 3.1. ALS

RAGE’s cytosolic tail binds the formin protein Diaph1, a critical interaction that transduces RAGE signaling; disrupting this RAGE-Diaph1 link abrogates downstream inflammatory signaling [41]. Thus, the RAGE-Diaph1 axis has emerged as a key mediator of cellular stress responses in the central nervous system. In this section, we delve into how RAGE, Diaph1, and associated ligands show altered expression in ALS and similar neurodegenerative diseases, drawing on transcriptomic, in vitro and in vivo, and proteomic evidence, with a focus on recent developments. Multiple studies indicate that RAGE and its pro-inflammatory ligands are upregulated in ALS.

In human ALS spinal cord tissue, RAGE mRNA and protein expression are significantly increased, accompanied by elevated levels of RAGE-binding ligands such as HMGB, S100B, and N-ε-carboxy-methyllysine (CML, an AGE) [15]. It was demonstrated via immunohistochemistry, qRT-PCR, and Western blots that ALS patient spinal cords have markedly higher RAGE, S100B, and HMGB1 compared to controls. These transcriptomic and proteomic changes suggest an overactive RAGE pathway in ALS, consistent with an inflammatory microenvironment.

Notably, ALS patients’ plasma levels of soluble RAGE (sRAGE, a decoy receptor) are lower, which correlates with more rapid disease progression. Low sRAGE may reflect consumption by excess ligands or a trait predisposing to ALS, and exogenous sRAGE has been proposed as a therapeutic to sequester ligand. Importantly, RAGE expression changes over the course of ALS and across cell types. In hSOD1G93A ALS mouse (a familial ALS model), RAGE and its ligand levels rise as the disease progresses. It was found that in SOD1 mice, HMGB1 and S100B protein expression peaks at symptom onset, supporting their role in early neuroinflammation [42]. Their transcriptomic analyses further showed RAGE, and these ligands are overexpressed in motor neurons, astrocytes, and microglia in both ALS mice and human patients [43]. These various cell types reveal an interconnected, RAGE-driven neuroinflammatory signaling in ALS. The activated microglia in the ALS spinal cord highly express RAGE and likely drive a self-propelled cycle of inflammation. RAGE activation in microglia induces the release of ROS and cytokines that upregulate RAGE and its ligands even more, further increasing motor neuron injury [43].

Transcriptomic profiling of ALS tissues confirms RAGE-axis dysregulation. An RNA-sequencing study of human ALS spinal cord found that *Ager* (RAGE gene) expression varies among patients. It inversely correlates with the age of disease onset. The higher the *Ager* expression, the earlier the ALS onset is, and its accelerated progression. Patients with high spinal cord RAGE levels showed gene expression changes in pathways related to extracellular matrix remodeling, lipid metabolism, and intercellular signaling. Worth noting, immunohistochemistry showed that microglia in high-*Ager* ALS cases have intense RAGE immunoreactivity, confirming observations in hSOD1G93A mice. These data suggest that elevated RAGE expression defines a molecular endotype of ALS with pronounced microglial activation and altered tissue homeostasis [43]. The pathogenic role of RAGE in ALS is strongly supported by loss-of-function experiments. Selective deletion of Ager in ALS models attenuates disease. Conditional knockout of RAGE in microglia of hSOD1G93A mice (induced at symptom onset) significantly extended survival and reduced neuroinflammation in male ALS mice.

Interestingly, many of the inflammatory gene expression changes seen in high-RAGE human ALS were normalized in RAGE-deficient microglial SOD1 mice, indicating that RAGE drives those transcriptomic programs [43]. Similarly, global RAGE knockout or pharmacological blockade delays ALS progression. A germline RAGE deficiency extended lifespan and dampened spinal cord inflammation in hSOD1G93A mice [44]. These in vivo findings link the transcriptomic signature of RAGE activation with actual disease-driving processes, and they highlight RAGE as a promising therapeutic target in ALS.

While Diaph1 has been less studied in ALS than RAGE, its role is implied by RAGE’s requirement for Diaph1 in signal transduction. Diaph1 is expressed in neural and myeloid cells; when RAGE is ligand-activated, the RAGE–Diaph1 complex triggers downstream pathways (MAPKs, NF-κB, Rho-GTPases, etc.) leading to inflammation. In vitro, siRNA knockdown of Diaph1 blocks RAGE–ligand signaling (preventing S100B- or AGE-induced kinase activation) without off-target effects [41]. Although direct transcriptomic data on Diaph1 in ALS are limited, one can infer that in the ALS spinal cord—where RAGE and ligands are elevated—Diaph1 is likely engaged. It may not be transcriptionally upregulated per se, but the functional coupling of RAGE and Diaph1 is active in ALS, propagating the inflammatory cascade.

Consistent with this idea, another study found that blocking the RAGE–Diaph1 interaction (using a small-molecule inhibitor) profoundly reduced inflammatory stress in diabetic mice [40]. Although that study addressed diabetes complications, it underscores a general principle applicable to ALS: Interrupting RAGE’s link to Diaph1 can mitigate downstream damage. Ongoing preclinical efforts are exploring such inhibitors in the context of ALS. In fact, the ALS Association recently funded development of a RAGE–Diaph1 antagonist for ALS therapy, reflecting the enthusiasm for this target.

### 3.2. AD

RAGE is strongly implicated in AD pathogenesis, and recent transcriptomic and histologic data suggest that the RAGE–Diaph1 axis is activated in AD brains. RAGE can bind amyloid-β (Aβ) peptides and transport them across the blood–brain barrier, and RAGE activation in microglia and neurons contributes to chronic inflammation and oxidative stress in AD [45]. There was an upregulated RAGE discovered in AD-affected brain regions [46]. For instance, RAGE is overexpressed in hippocampal neurons and microglia in AD [9].

Notably, Diaph1 expressions are also elevated in AD. It was reported that Diaph1 is highly expressed in aged human cortex and further upregulated in myeloid cells (microglia) during AD, with robust co-localization of Diaph1 and RAGE in activated microglia. Diaph1 upregulation in AD correlated with neutral lipid accumulation in those microglia. It links the RAGE–Diaph1 pathway to perturbed lipid metabolism and inflammation in AD [47]. These findings pointed out that chronic stimulation by Aβ and other RAGE ligands (like S100B or HMGB1 released from stressed cells) drives RAGE–Diaph1 signaling in AD microglia, promoting a pro-inflammatory and neurotoxic phenotype. Interfering with this axis may be beneficial.

Small-molecule RAGE inhibitors (such as TTP488 or FPS-ZM1) were shown to reduce brain Aβ load and neuroinflammation in AD-model mice [48]. Furthermore, in silico drug screens are now identifying novel RAGE antagonists as potential AD therapeutics. For example, a computational study screened >700,000 compounds and pinpointed several candidates with high affinity for RAGE’s ligand-binding domain. One of those compounds (“Hit-6”) presented stable binding in molecular dynamics simulations [48]. Such approaches underscore the growing interest in targeting RAGE–Diaph1 signaling to slow AD progression.

### 3.3. PD

Chronic neuroinflammation is also a feature of PD, and RAGE is upregulated in PD brains in association with disease severity [49]. RAGE is expressed on dopaminergic neurons, microglia, and endothelial cells in the nigra. Accumulating evidence implicates the RAGE and its obligatory signaling partner Diaph1 in the neuroinflammatory cascade that drives dopaminergic loss in Parkinson’s disease. In a unilateral 6-hydroxydopamine rat model, targeted intranigral delivery of the brain-penetrant RAGE antagonist FPS-ZM1 suppressed ERK1/2- and Src-mediated NF-κB activation. It curtailed microglial and astrocytic responses. It also reduced CSF and serum cytokines and preserved tyrosine-hydroxylase-positive neurons. Simultaneously, FPS-ZM1 ameliorated locomotor and exploration deficits. It indicates that RAGE–Diaph1 activity is an important upstream modulator of toxin-induced nigrostriatal degeneration [50].

Functionally, RAGE appears to mediate α-synuclein-driven inflammation: Extracellular α-synuclein aggregates can engage RAGE on microglia, triggering NF-κB and cytokine release. In vitro, blocking RAGE reduces microglial activation in response to α-syn fibrils [51]. In vivo, RAGE genetic deletion or silencing is protective in PD models. One study used siRNA to silence *Ager*/RAGE in a mouse PD model, which markedly ameliorated neuroinflammation and dopaminergic neurodegeneration. Mechanistically, RAGE silencing blunted the p38 MAPK and NF-κB pathways in the substantia nigra, reducing proinflammatory gene expression [52]. These data mirror the ALS findings and reinforce that RAGE (likely via Diaph1 signaling) orchestrates detrimental glial responses in PD.

Although specific data on Diaph1 in PD are limited, it is noteworthy that Diaph1 and RAGE were found to be co-expressed in myeloid cells of the healthy brain. This RAGE–Diaph1 co-localization is expected wherever RAGE signaling is active. Overall, the transcriptomic and experimental evidence in PD aligns with that in ALS. RAGE–Diaph1 activation skews microglia towards a pro-inflammatory, tissue-damaging state, and interrupting this axis is neuroprotective. RAGE overexpression and its inflammatory sequelae have been observed in other neurodegenerative or neuroinflammatory conditions as well. In multiple sclerosis (MS), RAGE and its ligand S100B are elevated at active demyelinating lesions, and patients with progressive MS show reduced monocyte surface RAGE (thought to reflect chronic ligand engagement and shedding). Interestingly, pharmacological RAGE blockade improved myelin repair in an MS model [53].

### 3.4. RAGE–Diaph1 Gene Differential Expression

Multiple high-throughput studies indicate that loss of the RAGE receptor alters gene expression profiles in a cell type and context-specific manner. For instance, in a diabetic atherosclerosis regression model, RAGE knockout profoundly affected lesional macrophage transcripts. RNA-seq of aortic plaque macrophages showed that interferon signaling pathways were significantly downregulated in diabetic mice lacking RAGE. Here, the master regulator *Irf7* was identified as a downregulated gene in Ager−/− macrophages, implicating RAGE in sustaining IRF7 and interferon-stimulated gene expression during plaque regression. These findings suggest that RAGE normally amplifies pro-inflammatory, interferon-related transcriptional programs in macrophages, which can hinder the resolution of diabetic vascular lesions. Deletion of the Diaph1 gene produces broad transcriptomic changes, especially in metabolic pathways. In a murine atherosclerosis model (Ldlr−/− mice on Western diet), global Diaph1 knockout resulted in 468 differentially expressed hepatic genes compared to Diaph1-intact controls [18]. Pathway analysis revealed a strong enrichment of lipid metabolism processes. For example, glycerophospholipid metabolism was among the top altered KEGG pathways [54]. Reactome and GO analyses likewise showed multiple lipid-related pathways (fatty acid catabolism, cholesterol transport) that were significantly dysregulated in Diaph1-deficient livers [18]. Consistent with these findings, specific genes governing lipid synthesis were suppressed by Diaph1 deletion. The transcripts of lipogenic enzymes (Acaca, Acacb, Gpat2, Fasn) and lipid droplet proteins (Lpin1, Lpin2) were downregulated in Diaph1−/− livers. These changes align with the physiological phenotype. Diaph1−/− mice showed reduced hepatic cholesterol and triglyceride accumulation and attenuated atherosclerosis progression [54]. It highlights a key role for Diaph1 in regulating metabolic gene networks linked to atherogenesis.

Emerging transcriptomic data highlight the RAGE–Diaph1 signaling axis as a critical node in diabetic tissue injury. Prolonged hyperglycemia alters gene expression in diabetic peripheral neuropathy (DPN). An RNA-seq study of diabetic mice found over 500 genes differentially expressed in the spinal cord. Pathway analysis showed the PI3K–Akt signaling axis (related to RAGE–Diaph1) as the most enriched pathway among the modified genes. The authors noted that DPN was associated with perturbations in RAGE–Diaph1 signaling in peripheral nerves, alongside these central nervous system transcriptomic changes [55]. This suggests that RAGE–Diaph1 driven signaling (via PI3K–Akt) may synergize with other molecular changes to promote diabetic neuropathy.

Clinical and translational data in humans also support the importance of this axis. In obese patients, gene-expression analyses of adipose tissue showed that RAGE and Diaph1 transcripts are co-regulated: In subcutaneous fat, *Ager* expression positively correlates with Diaph1 levels (and with the detoxifying enzyme GLO1), and this RAGE-axis signature correlates strongly with local inflammation, adipocyte dysfunction, and systemic insulin resistance (HOMA-IR). In fact, higher RAGE and Diaph1 expression in subcutaneous fat was linked to worse insulin sensitivity, underscoring an “immunometabolic” role of the AGE/RAGE/Diaph1 axis in human obesity-related insulin resistance [56].

## 4. AGER and DIAPH1 Gene Polymorphisms and Epigenetic Factors in Neurodegenerative Diseases

Neurodegenerative diseases such as ALS, AD, and PD are complex disorders with multifactorial and polygenic etiologies. Their development depends on interactions between inherited genetic variants and environmental as well as epigenetic factors. Increasing attention has been given in recent years to the analysis of single-nucleotide polymorphisms (SNPs), which may modulate the expression and function of proteins critical for neuronal homeostasis. Genetic variability can lead to increased expression of pro-inflammatory receptor isoforms or decreased expression of protective forms, translating into altered cellular responses to inflammatory stimuli [57,58,59,60]. One of the key pathways involved is the RAGE–Diaph1 axis, which plays a role in inflammatory responses, oxidative stress, and cytoskeletal remodeling in neural cells.

### 4.1. AGER

RAGE, encoded by the *AGER* gene, occurs in several isoforms, among which the soluble form—sRAGE—has been highlighted for its protective role. sRAGE lacks a transmembrane domain and is produced through alternative splicing (esRAGE) or proteolytic cleavage of the full-length membrane-bound receptor [61]. It acts as a molecular decoy, binding pro-inflammatory RAGE ligands (such as AGEs, HMGB1, and S100B), thereby preventing their interaction with the full receptor [62]. Numerous studies have shown that plasma and serum levels of sRAGE are significantly lower in patients with AD, vascular dementia (VAD), and mild cognitive impairment (MCI) compared to healthy controls, and lower sRAGE levels are associated with greater cognitive decline and disease severity [63,64]. Experimental models further demonstrate that increasing sRAGE, for example, through sRAGE-secreting mesenchymal stem cells, can reduce neuronal cell death, decrease amyloid-beta deposition, and suppress neuroinflammation, suggesting a therapeutic potential for sRAGE in conditions such as AD and Parkinson’s disease [37,38,39]. The reduction of sRAGE in neurodegenerative diseases may reflect an imbalance in the AGE–RAGE axis, contributing to chronic inflammation and neuronal damage [63,64]. Thus, sRAGE is not only a promising biomarker for early detection and progression of neurodegenerative disorders, but also a potential target for novel therapeutic strategies aimed at modulating neuroinflammatory processes [63,65,66].

Genetic variability within *AGER* may influence both RAGE expression levels and transcript isoform composition, as well as the receptor’s functional activity. Over 60 SNPs have been described within human *AGER*, several of which are considered functionally relevant [10]. The rs2070600 (Gly82Ser) polymorphism results in an amino acid substitution in the ligand-binding domain, increasing receptor affinity for AGEs and enhancing pro-inflammatory signaling. This variant may also alter receptor conformation, prolonging its activation and boosting recruitment of adaptor proteins and downstream signaling via NF-κB and MAPK pathways [67,68,69,70,71]. The SNP has been repeatedly associated with increased Alzheimer’s disease risk. The Ser82 allele was found to be significantly more frequent in AD patients than in controls, particularly among individuals lacking the APOE ε4 allele, suggesting an independent association with Alzheimer’s disease risk [72]. Moreover, carriers of the Ser82 variant had lower plasma sRAGE levels, supporting the idea that this polymorphism contributes to heightened inflammation by reducing sRAGE’s protective role [72].

Another *AGER* variant, rs1800625, may increase susceptibility to neurodegenerative diseases by amplifying inflammatory processes and disrupting RAGE-related signaling pathways. In a large Swedish population study, the rs1800625 risk allele was associated with increased dementia risk, indicating a potential role in neurodegenerative pathogenesis through inflammatory mechanisms and interactions with amyloid-β [73]. Furthermore, the genomic region encompassing *AGER* is functionally linked to the *NOTCH4* gene, pointing to complex interaction networks influencing dementia risk beyond classical immune pathways [73].

Regarding the rs1800624 variant of *AGER*, while direct evidence linking it to major neurodegenerative diseases is limited, some studies suggest it may modulate risk for neuroinflammatory and neurodegenerative disorders such as optic neuritis—a condition often preceding multiple sclerosis. A study in a Lithuanian population found no significant association between rs1800624 alone and optic neuritis risk; however, the A-G haplotype comprising rs1800624 and rs1800625 was significantly associated with increased risk, suggesting a possible synergistic effect in demyelinating disease pathogenesis [74]. Furthermore, rs1800624 has been linked to reduced risk of exudative age-related macular degeneration (AMD), a disease marked by chronic inflammation and retinal neurodegeneration. Carriers of the T allele had a significantly lower risk of developing exudative AMD, possibly indicating a protective role in chronic inflammation and oxidative stress-driven neurodegeneration [75].

### 4.2. DIAPH1

Similarly, the *DIAPH1* gene—which encodes protein diaphanous homolog-1, an essential transducer of intracellular RAGE signaling—shows considerable allelic and splice variant heterogeneity. *DIAPH1* variants have been implicated in neurodevelopmental and neurodegenerative disorders. Homozygous loss-of-function mutations in *DIAPH1* are linked to a syndrome involving microcephaly, epilepsy, cortical blindness, and developmental delay, highlighting its importance in CNS development [36,76,77]. Such mutations are also associated with mitochondrial dysfunction and impaired lymphocyte maturation, potentially contributing to neurological symptom progression [78]. Diaph1 regulates cytoskeletal structure and cell morphology essential for brain development [77]. Animal and human progenitor cell models confirm that *DIAPH1* dysfunction leads to defective neurogenesis and tissue organization [76].

Importantly, mutations in *DIAPH1* not only cause neurological symptoms but also immunological and metabolic dysfunction, underscoring the gene’s broad impact on cellular homeostasis [36]. In neurodegenerative contexts, observations of SCBMS (seizures, cortical blindness, microcephaly syndrome) suggest that *DIAPH1* loss-of-function leads to progressive brain degeneration. Cytoskeletal and mitochondrial disruptions may represent shared pathogenic mechanisms across various neurodegenerative disorders, in which DIAPH1 likely plays a disease-modifying role. Other *DIAPH1* allelic variants may affect both expression levels and functionality, e.g., altering interactions with FH1 or FH2 domains and impairing actin polymerization, directly impacting cytoskeletal stability. Some *DIAPH1* mutations may also affect its role as a Rho GTPase effector, influencing signaling via NF-κB, PI3K/Akt, and MAPK pathways—key regulators of inflammation and cell survival [79].

Neurodegenerative diseases, like many multifactorial disorders, result from multiple genes with small individual effects but synergistic pathological impact. Examples include known mutations in *SOD1*, *TARDBP*, *FUS*, and *C9orf72*, as well as subtler variants in *PFN1*, *VCP*, *ATXN2*, or *APOE* [25,26,27]. Genes involved in microglial function and inflammatory regulation, such as *TREM2* and *LRRK2*, also influence disease onset and progression, particularly in AD and PD [80,81]. Transcriptomic studies show that it is not only the expression of individual genes, but their network interactions and regulatory mechanisms, that determine disease pathophysiology.

### 4.3. AGER-DIAPH1 Epigenetics

Beyond polymorphisms, epigenetic factors—such as DNA methylation, histone modifications, and non-coding RNAs—play central roles in neuroinflammatory modulation, impacting expression of inflammation-related genes, including *AGER* and *DIAPH1*. Aging and environmental exposures (e.g., diet, stress, and infections) induce epigenetic changes that promote a chronic, low-grade inflammatory state in the brain, termed neuroinflammaging. This inflammation is considered a driving force behind AD and PD pathogenesis [82,83,84,85]. Promoter methylation of inflammatory genes like *AGER* may lead to silencing or overexpression, directly influencing CNS inflammatory intensity. Histone modifications (e.g., acetylation and methylation) regulate chromatin accessibility and the expression of pro-inflammatory and neuroprotective genes [83,84].

Although direct data on *DIAPH1* epigenetic regulation are limited, its key role in RAGE signaling suggests its function may be modulated by upstream epigenetic changes affecting *AGER* or other inflammatory mediators [82,83].

Importantly, epigenetic modifications are reversible, making them attractive targets for therapeutic intervention in neurodegenerative disease. Restoring normal DNA methylation or histone modification profiles could help mitigate chronic inflammation and slow neurodegenerative progression [83]. Together, genetic variability, alternative splicing, and epigenetic regulation compose a multidimensional framework of neurodegenerative disease pathogenesis. Understanding these mechanisms in the context of *AGER* and *DIAPH1* may not only help identify at-risk individuals but also support the development of personalized therapies targeting the RAGE–Diaph1 axis to restore neuroimmune balance in the CNS.

## 5. RAGE Signaling Inhibition as a Potential ALS Treatment—Lessons from Our Studies

Our earlier experiments support the notion that RAGE signaling plays a significant role in the progression of neurodegeneration, specifically in ALS and diabetic peripheral neuropathy. We have shown that RAGE and its ligands are highly expressed in the human and mouse ALS spinal cord [15]. Furthermore, experiments with hSOD1G93A mice treated with either soluble RAGE (sRAGE), a 40 kDa recombinant protein that acts as a RAGE decoy suppresses RAGE–ligand binding or vehicle, murine serum albumin (MSA) revealed that sRAGE-treated hSOD1G93A transgenic mice had extended survival, improved motor function performance and higher neuronal counts compared to control (MSA)-treated mice [86]. In another study, published in 2021, we delved deeper into the role of RAGE and its signaling pathways, demonstrating that microglial RAGE exacerbates the progression of neurodegeneration in hSOD1G93A mice in a sex-dependent manner. We also found that AGER expression varied relative to differentially expressed pathways related to lipid metabolism, intracellular communication, and extracellular matrix regulation, and its levels negatively correlated with the age at onset and age at death or tracheostomy in ALS patients’ cervical spinal cords [43].

**Figure 3:**
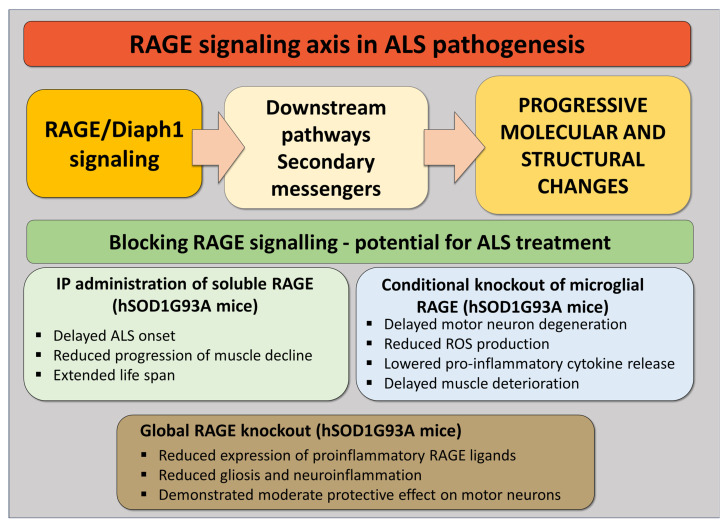
RAGE in ALS as both a factor involved in ALS pathogenesis as well as a potential target in its treatment.

Our most recent studies on RAGE involvement in ALS highlighted that, even at the early stages of the disease, levels of the neuroprotective sRAGE are reduced, while levels of AGEs and AOPPs (advanced oxidation protein products) are increased in the blood samples of ALS patients [87]. Similarly, we demonstrated that hSOD1G93A mice devoid of RAGE exhibit better motor performance and, on average, live longer compared to their counterparts, warranting further investigation into the role of RAGE and its ligands in the progression of ALS [44]. Finally, examining the role of RAGE–Diaph1 inhibition in neurological complications of diabetes has demonstrated that diabetic mice with simultaneous deletion of *AGER* and *DIAPH1* display slower rates of sciatic nerve degeneration, further validating the importance of studying the RAGE signaling axis in the pathogenesis of neurodegenerative diseases (Figure 3).

## 6. Limitations and Challenges in Targeting the RAGE–Diaph1 Pathway

Although numerous studies emphasize the pathogenic role of RAGE–Diaph1 signaling in neurodegenerative diseases, several investigations report contradictory, null, or even detrimental effects of targeting this pathway. Integrating these findings offers a more balanced perspective on its therapeutic potential.

Animal model studies have shown that complete RAGE ablation does not always produce neuroprotection and, in certain contexts, can worsen disease outcomes. This may be partly because RAGE performs important physiological functions, including roles in immune regulation, tissue repair, and cellular homeostasis, and its complete loss can impair these beneficial processes. In hSOD1G93A ALS mice, pharmacological RAGE inhibition improved muscle function and reduced neuroinflammation but failed to delay onset or extend survival, while complete genetic knockout of RAGE shortened lifespan [88]. Similarly, in an experimental autoimmune encephalomyelitis model, RAGE-deficient mice exhibited disease severity comparable to wild-type controls, whereas treatment with soluble RAGE decoy protein markedly reduced inflammation and clinical signs [89]. These results suggest that RAGE ligands may continue to drive pathology through alternate receptors such as TLRs when RAGE is absent, and that total blockade of RAGE activity can deprive the organism of its normal protective functions.

Contradictory findings are also seen in Alzheimer’s disease research. In transgenic ArcAβ mice, RAGE deletion did not reduce amyloid burden, gliosis, or cognitive decline [90]. Clinical translation has been challenging: A Phase 2 trial of the RAGE antagonist azeliragon (TTP488) revealed a narrow therapeutic window, with high-dose treatment causing cognitive worsening and low-dose treatment producing only modest benefit [91]. The subsequent large Phase 3 STEADFAST trial in mild AD failed to demonstrate any cognitive or functional advantage over placebo [92], highlighting the complexity of human disease and possible compensatory mechanisms.

Additional complexities arise from context-dependent effects. For example, selective microglial RAGE deletion in ALS extended survival in male mice but had no effect in females, indicating sex-dependent responses [43]. This suggests that biological factors such as sex, disease stage, and cell-type specificity may influence therapeutic efficacy.

Collectively, these findings caution against indiscriminate inhibition of RAGE–Diaph1. Key considerations for future strategies include the potential need for partial rather than complete blockade, dual targeting of RAGE and its ligands, careful patient stratification, and biomarker-guided therapy to ensure optimal target engagement. Such an approach acknowledges the mixed preclinical and clinical outcomes to date, while still recognizing the pathway’s potential as a disease-modifying target.

## 7. Conclusions

Beyond a well-known role in metabolic and inflammatory disorders, the RAGE–Diaph1 axis manifests in maladaptive responses within the CNS. Transcriptomic and proteomic evidence point to it as a contributor in conditions such as ALS, AD, PD, and related disorders. This ongoing stimulation heightens glial reactivity. This further destabilizes neuronal homeostasis and is closely associated with earlier disease onset and more rapid progression. The pathway converges on core intracellular signaling networks, including MAPK, NF-κB, and PI3K–Akt, leading to combined inflammatory, metabolic, and structural disturbances. Functional inhibition of this axis through genetic deletion of AGER or DIAPH1, administration of soluble RAGE, or disruption of their protein-to-protein interaction has been shown in preclinical models to reduce microglial overactivation. It leads to preservation of neuronal populations and extends survival. Together, these findings establish RAGE–Diaph1 not only as a marker of neurodegenerative pathology but as a central, disease-modifying mechanism. Deeper characterization of its cell-type-specific roles, temporal activation patterns, and interaction networks may lead to more precise therapeutic approaches. Combining molecular inhibition with biomarkers to monitor target engagement could open new possibilities of interventions across neurodegenerative disorders.

## Data Availability

No new data were created or analyzed in this study. Data sharing is not applicable to this article.

## Abbreviations

The following abbreviations are used in this manuscript:

ALS	Amyotrophic lateral sclerosis
RAGE	Receptor for advanced glycation end-products
AGEs	Advanced glycation end-products
ROS	Reactive oxygen species
AD	Alzheimer’s disease
PD	Parkinson’s disease
HD	Huntington’s disease
HMGB1	High mobility group protein B1
MS	Multiple sclerosis
PPR	Pattern recognition receptor
DAMPs	Damage-associated molecular patterns
mRAGE	Membrane-bound RAGE
sRAGE	Soluble RAGE
SCA1	Spinocerebellar ataxia type 1
DRFs	Diaphanous-related formins
CML	N-ε-carboxy-methyllysine
AMD	Age-related macular degeneration
TLR	Toll-like receptor
